# Improved injection site reactions after switching from adalimumab reference to adalimumab biosimilar LBAL for ulcerative colitis: A case report

**DOI:** 10.1097/MD.0000000000040400

**Published:** 2024-11-01

**Authors:** Yudai Hirakawa, Toshihiko Kakiuchi, Masato Yoshiura, Nao Kikkawa

**Affiliations:** a Department of Pediatrics, Faculty of Medicine, Saga University, Saga, Japan; b Department of Pharmacology, Saga University Hospital, Saga, Japan.

**Keywords:** adalimumab biosimilar, adalimumab reference, injection site reaction, LBAL, medical switch

## Abstract

**Rationale:**

Adalimumab (ADA) is an antitumor necrosis factor agent that is used for the treatment of inflammatory bowel disease. However, its cost has resulted in varying degrees of restricted access across global healthcare economies. Biosimilars are agents that contain a similar version of the active substance of an already approved original biologic agent and are intended to be used for the same indication as the reference product. In general, biosimilars follow the originator; therefore, information on its efficacy and safety had been few. Some studies have reported on replacement of the originator with a biosimilar of the same efficacy because of medical reasons.

**Patient concerns:**

A 15-year-old girl with steroid-dependent ulcerative colitis that relapsed after vedolizumab was treated with ADA reference. Six weeks after starting ADA reference, her gastrointestinal symptoms had completely resolved, however, immediately after the eighth dose of ADA reference, redness, swelling, and pruritus were noted at the injection site on the left thigh.

**Diagnosis:**

Allergic reaction caused by the ADA reference.

**Intervention:**

ADA reference was changed to ADA biosimilar LBAL.

**Outcomes:**

ADA biosimilar LBAL was continued without any symptoms, such as local swelling, redness, or itching. In addition, there was no deterioration of gastrointestinal symptoms.

**Lessons:**

We showed the efficacy and safety of ADA biosimilar LBAL as an alternative to ADA reference, which caused injection site reactions. Changing from ADA reference to ADA biosimilar because of adverse events may be an option that needs careful observation, considering that the originator and the biosimilar are not exactly the same.

## 1. Introduction

Adalimumab (ADA) is an antitumor necrosis factor (TNF) agent that is used in the treatment of inflammatory bowel disease (IBD), which includes Crohn disease and ulcerative colitis (UC). In pediatric UC, infliximab is recommended when the disease is uncontrollable by 5-aminosalicylic acid (5-ASA) and azathioprine, chronically active, or corticosteroid-dependent.^[1,2]^ In those who relapse or are intolerant to infliximab, based on serum levels and antibodies, ADA could be considered and is convenient for school-age children, because it can be injected at home. Notably, citrate as an excipient in ADA has been associated with pain at the injection site.^[3]^ After the introduction of a new citrate-free ADA formulation in 2016, the priority of selecting ADA orders has increased, especially for children who are vulnerable to pain. Consequently, improvement of adherence was expected.

Over the last 2 decades, biological TNF inhibitors, such as ADA, have led to achievable outcomes in patients with a wide variety of immune-mediated inflammatory diseases, including rheumatoid arthritis, axial spondyloarthropathy, and IBD. However, the high cost of ADA has resulted in varying degrees of restricted access across global healthcare economies.^[4]^ Biosimilars are agents that contain a similar version of the active substance of an already approved original biologic agent and are intended to be used for the same indication as the reference product.^[5]^ In October 2018, expiration of the patent for the originator ADA molecule allowed the availability of new biosimilar medications.^[5]^ In Japan, 3 ADA biosimilars are covered by the public health insurance for adult UC. According to the European Crohn's and Colitis Organization Position Statement,^[6]^ biosimilars are not fully comparable with the generic molecules. Therefore, choosing a biosimilar over an established biologic agent to save costs could be accompanied by several issues. In general, biosimilars follow the originator; therefore, information on its efficacy and safety had been few. Many studies have examined the safety of switching from the originator to biosimilars for nonmedical reasons.^[7]^ On the other hand, only few studies have reported on replacement of the originator with a biosimilar of the same efficacy because of medical reasons. In this report, we presented a pediatric UC case, in which injection site reactions to ADA reference were improved by switching to ADA biosimilar LBAL.

## 2. Case report

**Figure 1.**
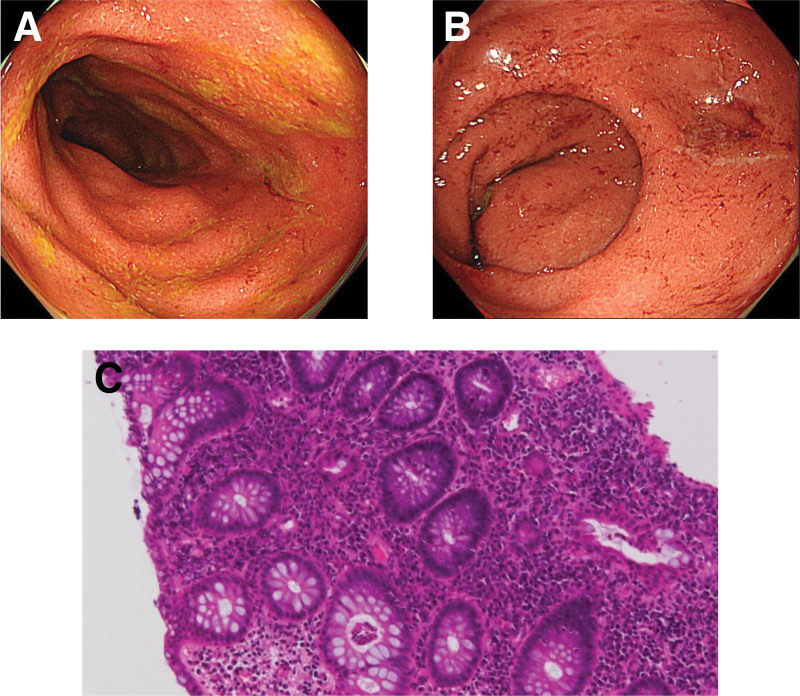
Total colonoscopy and pathological findings at the time of ulcerative colitis diagnosis. From (A) the ascending colon to (B) the rectum, the mucous membrane is reddish and edematous and bleeds easily. (C on hematoxylin-eosin stain) On pathological examination, there are mucosal erosion, degeneration, and epithelial regeneration; edema, hyperemia, and inflammatory cell infiltration in the lamina propria; and diffuse cryptitis and crypt abscesses.

A 15-year-old girl presented to our hospital with diarrhea and bloody stool for 1 month and consequent weight loss. On admission, physical examination revealed the following: height, 160.5 cm (standard deviation +0.7); weight, 42.3 kg (standard deviation −0.6), which was 2.8 kg less than the weight 1 month ago; body temperature, 36.3°C; heart rate, 72 beats per minute; and blood pressure, 96/54 mm Hg. She had increased bowel sounds. Complete blood count revealed the following: white blood cell count, 7400 (normal range [NR]: 7000–15,000) cells/mL; hemoglobin, 12.9 (NR: 13.7–16.8) g/dL; and platelet count, 245 × 10^3^ (NR: 158–348 × 10^3^) cells/mL. Her laboratory test findings were as follows: total protein, 7.0 (NR: 6.8–8.1) g/dL; albumin, 4.0 (NR: 4.1–5.1) g/dL; C-reactive protein, 0.08 (NR: <0.14) mg/dL; serum amyloid A protein, 1.7 (NR: <8.0) µg/mL; and erythrocyte sedimentation rate, 18 (NR: <17) mm/H. No infection was detected in the stool culture, and the fecal calprotectin level was 5620 (NR: <50) mg/kg. Stool adenovirus, rotavirus, and norovirus antigen tests were negative. Based on the symptoms that were indicative of UC, total colonoscopy was performed and showed reddish mucous membrane that bled easily and edema from the distal side of the ascending colon to the rectum (Fig. 1A and B). On pathological examination, there were mucosal erosion, degeneration, and epithelial regeneration; edema, hyperemia, and infiltration of inflammatory cells (i.e., lymphocytes, plasma cells, and neutrophils) in the lamina propria; and diffuse cryptitis and crypt abscesses (Fig. 1C). Given these findings, the patient was diagnosed as UC, with an Ulcerative Colitis Endoscopic Index of Severity score of 5 and a Pediatric Ulcerative Colitis Activity Index (PUCAI) score of 55.

**Figure 2.**
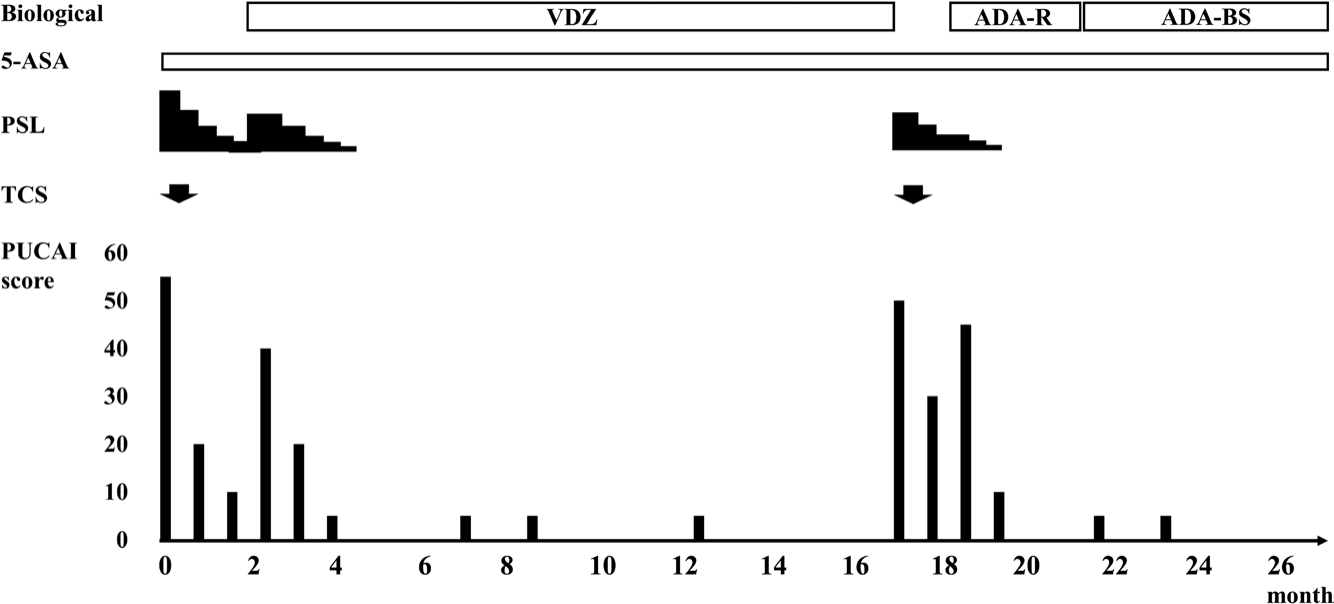
The patient’s clinical course and treatment for ulcerative colitis. A 15-yr-old girl presented to our hospital with diarrhea and bloody stool for one month and consequent weight loss. Based on the TCS findings, she was diagnosed as UC, which was steroid-dependent. VDZ administration initially afforded remission. However, after 15 mo, VDZ was changed to ADA reference because of UC relapse. On the eighth and ninth ADA reference administration, rash, swelling, and itching appeared at the injection site on the anterior left thigh. After switching to ADA biosimilar LBAL, there were no injection site reaction and no exacerbation of UC. 5-ASA = 5-aminosalicylate acid, ADA = adalimumab, ADA-BS = adalimumab biosimilar, ADA-R = adalimumab reference, PSL = prednisolone, PUCAI = pediatric ulcerative colitis activity index, TCS = total colonoscopy, UC = ulcerative colitis, VDZ = vedolizumab.

As shown in Figure 2, she was initially treated with 5-ASA and 1 mg/kg prednisolone (PSL), which improved her symptoms. However, her gastrointestinal symptoms soon recurred as the dose of PSL was reduced. We diagnosed her condition as steroid-dependent refractory UC, for which the highly safe vedolizumab (VDZ) was introduced 2 months after,^[8]^ along with increase in the PSL dose. Thereafter, her symptoms improved rapidly, and the PSL dose was reduced without recurrence of symptoms. Fifteen months after starting VDZ, she began to experience abdominal pain, diarrhea, grossly bloody stools, and increased stool frequency. Her PUCAI score deteriorated from 0 to 50. Blood tests, rapid antigen tests, and stool cultures ruled out bacterial and viral infections. Repeat total colonoscopy confirmed relapsed UC (Ulcerative Colitis Endoscopic Index of Severity score, 4). While continuing VDZ, 1 mg/kg PSL was resumed, and the symptoms gradually improved. However, the gastrointestinal symptoms worsened as the PSL dose was reduced. Judging that the therapeutic effect of VDZ had weakened, ADA reference was introduced at an initial dose of 160 mg and subsequent doses of 80 mg each after 1 week and every 2 weeks thereafter. ADA reference was injected subcutaneously on the anterior surface of the thigh, alternating on the left and right for each dose.

**Figure 3.**
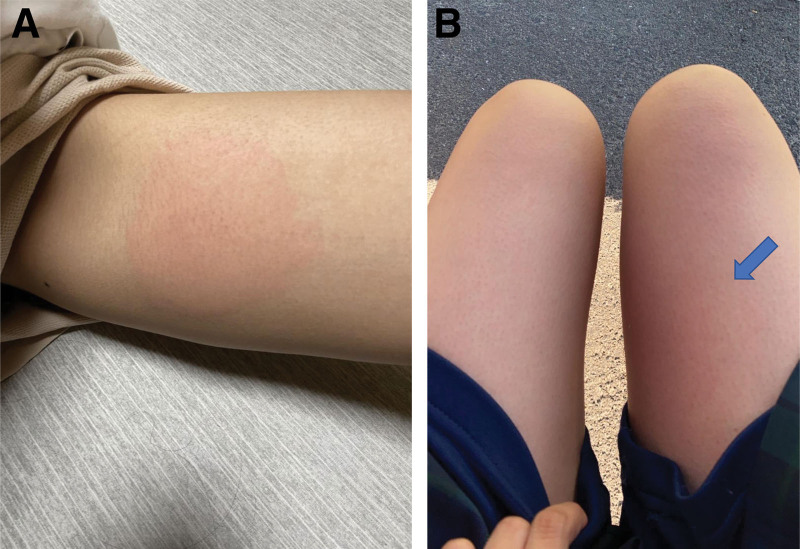
Injection site reactions to ADA-R. (A) Two hours after the eighth administration, a wheal formation (i.e., redness and swelling) is noted at the injection site on the anterior surface of the left thigh. (B) After 24 h of the ninth administration, there is recurrent and more severe wheal formation at the injection site on the right thigh. ADA-R = adalimumab reference.

Six weeks after starting ADA reference, her gastrointestinal symptoms had completely resolved (PUCAI score, 5), and she continued receiving ADA reference without obvious adverse events. However, immediately after the eighth dose of ADA reference, redness, swelling, and pruritus were noted at the injection site on the left thigh (Fig. 3A). This symptom completely improved the next day. At the ninth dose, the symptoms recurred and were more severe at the injection site on the right thigh (Fig. 3B). The localized redness and swelling resolved in 2 days, but the pruritus persisted for 5 days before spontaneous resolution. Judging the symptoms as an injection site allergic reaction, ADA reference was changed to ADA biosimilar LBAL, which was administered at a dose of 80 mg on the anterior surface of the thigh every 2 weeks, alternately on the left and right. ADA biosimilar LBAL was continued without any symptoms, such as local swelling, redness, or itching. In addition, there was no deterioration of gastrointestinal symptoms. Six months after changing to ADA biosimilar LBAL, the UC remained stable without injection site reaction.

## 3. Discussion

Unlike conventional medications, biosimilars are not considered to be completely equivalent to their biological originator, because they are large and complex molecules that are extremely sensitive to any slight change in the manufacturing process.^[9]^ However, biosimilars have been encouraged as a more affordable therapeutic alternative that may alleviate the financial burden on the healthcare system.^[10]^ LBAL is one of the ADA biosimilars that were developed and manufactured by LG Chem using Chinese hamster ovarian cells. A comparative assessment of physicochemical and biological attributes demonstrated that LBAL was structurally and functionally similar to ADA reference, thereby, supporting its biosimilarity in clinical efficacy and safety.^[11]^

The clinical course of this UC case demonstrated the efficacy and safety of switching from ADA reference to ADA biosimilar LBAL for a medical reason (i.e., injection site reaction to ADA reference). Injection site reactions are common with subcutaneously administered anti-TNF agents, and were reported to occur at a frequency of 20% with ADA.^[12]^ An immunoglobulin E-mediated immediate type I hypersensitivity reaction was reported to mediate worsening injection site reactions in some patients who received ADA.^[13]^ Table 1 shows a list of the excipients in ADA reference and ADA biosimilar LBAL. D-mannitol is an excipient in ADA reference but not in ADA biosimilar LBAL. Therefore, the injection site reactions in the present case were probably secondary to an allergic reaction to D-mannitol rather than to ADA itself. However, in one report, intradermal test with D-mannitol was negative in all patients who had injection site reactions to ADA or etanercept.^[14]^

**Table 1:** Comparison of excipients found in the adalimumab reference and adalimumab biosimilar LBAL.

Adalimumab reference (syringe and autoinjector)	Adalimumab BS LBAL (syringe and autoinjector)
Adalimumab 20, 40, and 80 mg	Adalimumab 20, 40, and 80 mg
Polysorbate 80 (0.2, 0.4, 0.8 mg)	Polysorbate 80 (0.2, 0.4, 0.8 mg)
D-mannitol (8.4, 16.8, 33.6 mg)	
	L-Arginine hydrochloride (2.11, 4.21, 8.42 mg)
	L-Methionine (0.15, 0.3, 0.6 mg)
	Sucrose (11, 22, 44 mg)

BS = biosimilar.

Infusion-related reactions (IRRs) to biological agents are serious safety concerns in the management of IBD. However, IRRs include various types of adverse reactions and are thought to have the following possible causes: complement activation-related pseudo-allergy; cytokine release syndrome (CRS); immunoglobulin E-mediated anaphylaxis (severe) or allergic reactions (mild-moderate), including cross-reactivity; or anaphylactoid reaction.^[15]^ The first 2 lead to true IRRs, whereas the latter 2 are allergic-related reactions to biologic agents.^[16]^ In the present case, complement activation-related pseudo-allergy and CRS were unlikely causes of the IRR, because these frequently occur on the first administration and are less frequent after the second administration.^[17]^ The injection site reactions in the present case were suspected to be caused by an allergic reaction to D-mannitol, because these appeared after 8 times of ADA reference administration. However, tests, such as the intradermal test, could not be carried out to confirm this suspicion. In addition, because biosimilars are not considered to be completely equivalent to their biological originator, it may have been an allergic reaction caused by the ADA component of the originator.

In general, occurrence of adverse events with a biological originator would lead to reluctance in switching to a biosimilar agent with the same ingredients. This is because there is more information on the safety of originators than of biosimilars.^[18]^ However, in the present case, the adverse events resolved after switching to a biosimilar with the same ingredients. Pediatric IBD is highly severe and inevitably lasts for a long period.^[19]^ Therefore, carefully switching from a biological originator to a biosimilar agent may be considered in order to continue treatment.

In conclusion, changing from ADA reference to ADA biosimilar for medical reasons, such as adverse events, may be an option under careful observation, considering that the originator and the biosimilar are not exactly the same.

## Acknowledgments

The authors thank the patient’s family for providing consent and granting permission to draft and publish this case report.

## Author contributions

**Conceptualization:** Yudai Hirakawa, Toshihiko Kakiuchi.

**Data curation:** Yudai Hirakawa, Toshihiko Kakiuchi, Masato Yoshiura.

**Investigation:** Yudai Hirakawa, Toshihiko Kakiuchi, Nao Kikkawa.

**Methodology:** Yudai Hirakawa, Toshihiko Kakiuchi.

**Writing – original draft:** Yudai Hirakawa, Toshihiko Kakiuchi.

**Formal analysis:** Toshihiko Kakiuchi.

**Project administration:** Toshihiko Kakiuchi.

**Supervision:** Toshihiko Kakiuchi, Nao Kikkawa.

**Validation:** Toshihiko Kakiuchi, Nao Kikkawa.

**Visualization:** Toshihiko Kakiuchi.

**Writing – review & editing:** Toshihiko Kakiuchi, Nao Kikkawa.
